# Influence of Wood-Based Biomass Ash Admixing on the Structural, Mechanical, Hygric, and Thermal Properties of Air Lime Mortars

**DOI:** 10.3390/ma12142227

**Published:** 2019-07-10

**Authors:** Milena Pavlíková, Lucie Zemanová, Jaroslav Pokorný, Martina Záleská, Ondřej Jankovský, Michal Lojka, Zbyšek Pavlík

**Affiliations:** 1 Department of Materials Engineering and Chemistry, Faculty of Civil Engineering, Czech Technical University in Prague, Thákurova 7, CZ-166 29 Prague, Czech Republic; 2 Department of Inorganic Chemistry, Faculty of Chemical Technology, University of Chemistry and Technology, Technická 5, 166 28 Prague 6, Czech Republic

**Keywords:** wood-based biomass ash, industrial waste, lime-pozzolan renovation mortars, pozzolanic activity, binder physical and chemical analyses, functional properties, environmental assessment

## Abstract

Mechanically-activated wood-based biomass ash (WBA) was studied as a potential active admixture for design of a novel lime-pozzolan-based mortar for renovation purposes. The replacement ratio of lime hydrate in a mortar mix composition was 5%, 10%, and 15% by mass. The water/binder ratio and the sand/binder ratio were kept constant for all examined mortar mixes. Both binder constituents were characterized by their powder density, specific density, BET (Brunauer–Emmett–Teller), and Blaine specific surfaces. Their chemical composition was measured by X-ray fluorescence analysis (XRF) and mineralogical analysis was performed using X-ray diffraction (XRD). Morphology of WBA was investigated by scanning electron microscopy (SEM) and element mapping was performed using an energy dispersive spectroscopy (EDS) analyzer. The pozzolanic activity of WBA was tested by the Chapelle test and assessment of the Portlandite content used simultaneous thermal analysis (STA). For the hardened mortar samples, a complete set of structural, mechanical, hygric, and thermal parameters was experimentally determined. The mortars with WBA admixing yielded similar or better functional properties than those obtained for traditional pure lime-based plaster, pointing to their presumed application as rendering and walling renovation mortars. As the Chapelle test, STA, and mechanical test proved high pozzolanity of WBA, it was classified as an alternative eco-efficient low-cost pozzolan for use in lime blend-based building materials. The savings in CO_2_ emissions and energy by the use of WBA as a partial lime hydrate substitute in mortar composition were also highly appreciated with respect to the sustainability of the construction industry.

## 1. Introduction

The air-hardening lime in conjunction with naturally occurring pozzolanic materials was until the 1850s the principal binder for use in mortars and other building components [1]. Later, synthetic hydraulic limes found use based on extensive research on the hydraulic properties of lime-pozzolan binders, starting at the end of the 18th century. After the first invention of cement in the first half of the 19th century, cement became the prevailing binder of the building industry due its mechanical strength and rapid setting [2,3].

As lime was used for rendering and structural purposes in building construction for centuries, most of the historical and old buildings were constructed by the use of lime, enriched deliberately or randomly by different pozzolanic materials [4,5,6,7]. Nowadays, the use of lime-based mortars is highly reduced due to the widespread application of PC-based materials possessing high strength, durability, corrosion resistance, and other advantageous properties. On the other hand, some damage to historical buildings associated with the structural, hygrothermal, and other building pathology problems are known to have been caused by the use of cement in repair and restoration works, which highlighted the incompatibility of water vapor impermeable and rigid cement matrix with its historic lime-based counterparts [8,9,10]. Compared to lime-based materials, PC materials possess significantly lower water vapor transmission rates. It is recognized that the high mechanical resistance of cement-based materials is unnecessary in many applications, e.g., in structures whose inbuilt materials have significantly lower strength than Portland cement. Cement also exhibits other adverse properties; it has a much higher thermal expansion coefficient than most masonry materials and it can contain soluble salts that leach out over time and evoke disruptive crystallization pressures and form unaesthetic salt crystals on the building surface [11]. Based on a given rationale, it is evident that the use of PC in the conservation and renovation of historical buildings is a wrong choice. 

The cultural heritage authorities put emphasis on the application of compatible materials in repair and renewal of historically-valuable buildings. When old heritage buildings are repaired, we must bear in mind not only the functional aspects of inbuilt materials but also the preservation of their history [12]. In this respect, an adequate choice of repair mortars is crucial for the success of a restoration intervention [13,14]. It is generally accepted that mortar should be more permeable for water vapor than the other masonry constituents in order to eliminate their possible damage by coupled moisture–salt action as high permeability of renovation mortar allows water evaporation before it spreads throughout the whole structure [9]. Similarly, pointing and structural mortars must have lower mechanical strength than walling stone-blocks and bricks to accommodate possible slight movements of the building. To meet the above given requirements on compatibility and functional parameters of repair materials, lime-based mortars have been increasingly used for this purpose [15,16,17], and new air lime mortars based on the study of old treatises [18] and analysis of historical mortars were developed [19,20]. In the last three decades, intensive research on air lime-based mortars was conducted aiming above all at the nature, quality, and proportions of mortar components, conditions of lime production and its characteristics, environmental weathering, parameters affecting carbonation and formation of a porous structure, application processes, etc. The results coming from air lime mortar investigations were summarized, for example, in a comprehensive review published by Veiga [21] who referenced more than 100 scientific papers aimed at analysis, design, and testing of historical and recent lime-based mortars. Veiga concluded that lime-based mortars are the most suitable for conservation of old buildings, but their use is still rare in conservation interventions and repairs of the buildings that were originally lime-based [21]. In this sense, one must consider also obvious disadvantages of these mortars, such as slow setting and hardening and thus low early strength, susceptibility to water, salt, and frost action. As mortars for renovation purposes must be sufficiently durable to withstand environmental weathering, blended mortars are a promising alternative to combine the advantages of both air lime and hydraulic binders and pozzolans. As use of PC in restoration of historical buildings is often forbidden by cultural heritage authorities, hydraulic lime-based mortars and lime-pozzolan-based mortars represent a promising solution from the point of view of their technical parameters and compatibility with authentic materials. A number of binary and ternary lime-pozzolan mortars were developed until now based on the use of various pozzolanic materials that react with calcium hydroxide in the presence of water to form hydraulic products similar to those obtained by hydration of PC. Both natural and artificial pozzolans were reported as economically and environmentally advantageous materials for lime blends. Among natural pozzolans used for the improvement of durability and strength parameters of lime mortars belong zeolite [22,23,24], volcanic ash [25,26], pumice [27], diatomite [28,29], etc. Artificial pozzolans usually come from thermal processing (metakaoline [30,31], perlite [32]), coal and agricultural product combustion (coal fly ash [33], palm oil ash [34,35], sugar cane bagasse ash [36], rice husk ash [37]), calcination of different types of clays, or are in the form of industrial by-products (ground ceramic waste [38], brick powder [39], silica fume [40], ground granulated blast furnace slag [41], cement kiln dust [42], waste graphite powder [43], etc.). As most of these pozzolanic materials found are already used in PC-based concrete manufacturing that consumes large quantities of natural as well as artificially produced materials, there is a need to look for further active mineral admixtures that could potentially improve properties of lime-based materials, especially for those that are low-cost, renewable, available in huge quantities, and have no better usage both from the economic and environmental point of view. Due to the uncontrolled exhaustion of natural resources by the building industry, sustainable pozzolanic materials that were formerly considered as waste must be investigated in this respect.

Today’s increased interest in the environmental sustainability and production of renewable energy leads worldwide to the combustion of huge quantities of biomass that is responsible for approximately 480 million tons of ashes generated annually [44]. In respect to the Directive 2009/28EC of the European Parliament and Council [45] that dictates European member states to produce a minimum of 20% of the total generated energy renewably in 2020, growing quantities of biomass ashes can be anticipated in the near future. As biomass ashes have similar properties to fly ashes coming from coal combustion, their applicability as supplementary cementitious materials has been increasingly studied in recent years [46]. An overwhelming majority of reported studies was aimed at the use of biomass ash in PC-based blends [47,48,49]. These papers reported on proper chemical and mineralogical composition of different types of biomass ashes, their high specific surface, particle size distribution similar to that of cement, and high pozzolanity. On the other hand, the use of biomass ashes as pozzolans for air lime-based materials was studied only scantly up to now. Therefore, wood-based biomass ash was studied in this paper, as an alternative pozzolan for production of lime-based mortars for repair applications. For mortars with biomass ash used as an active mineral admixture, a unique set of structural, mechanical, hygric, and thermal properties was experimentally obtained, allowing assessment of the applicability of the developed mortars for construction and rendering purposes.

## 2. Experimental 

Within the extensive experimental campaign, lime-based mortars with partial lime hydrate (LH) replacement with wood-based biomass ash (WBA) were developed and tested. First, detailed characterization of both binders (WBA, LH) was done. It comprised their basic physical characterization, assessment of chemical and mineralogical composition, morphology analysis, particle size distribution measurement, and pozzolanic activity testing. The obtained data served as basic input information for mortar mix design and choice of the rate of LH replacement. The mortar samples were matured for 28 days and 365 days, respectively. The tests of the hardened mortar samples included the experimental assessment of their basic structural properties, mechanical properties, hygric properties, thermal properties, pore size distribution, and simultaneous thermal analysis (STA). Finally, the environmental benefits of the use of WBA as an LH substitute were evaluated based on calculation of savings in energy consumption and CO_2_ emissions for production of LH–WBA blends.

### 2.1. Materials and Design 

The wood-based biomass ash was obtained from the Plzeň heating plant, Czech Republic. It is the heating plant for biomass combustion that annually combusts approximately 170 kt of biomass yielding approximately 10 kt of bottom ash. The WBA is a by-product of wood chip combustion. The wood chips were composed of approximately 89% of softwoods (spruce 70%, pine 22%, larch 5%, other evergreens 3%) and 11% of hardwoods (oak 28%, beech 28%, birch 13%, other broadleaves 31%). It was mechanically activated by milling in a vibratory disc mill to a granularity similar to LH. 

The WBA was used in a lime-based mortar mix composition as a partial LH substitution. Prior to use, the ash was dried in a vacuum oven at 70 °C for 72 h. The substitution ratio was 5 wt % (MWBA 5), 10 wt % (MWBA 10), and 15 wt % (MWBA 15) of LH. The mortars were prepared using lime hydrate LH CL90-S as the primary binder. It was produced according to the standard EN 459-1 [50] in lime kiln Čertovy Schody, a.s, Czech Republic, member of the Lhoist group. Silica sand of particle size fraction 0–2 mm (Filtrační písky s.r.o., Chlum u Doks, Czech Republic) was mixed from three normalized sand fractions 0–0.5 mm, 0.5–1 mm, and 1–2 mm. The weight ratio of particular sand fractions was 1:1:1. The water/binder ratio of 1.0 and the sand/binder (LH + WBA) ratio of 3.0 were constant for all mortar mixes. For the evaluation of the effect of WBA incorporation in mortar mix on material properties, samples of reference lime mortar (MR) were prepared and tested according to the standards EN 10115-2 [51] and EN 196-1 [52]. The detailed composition of studied mortar mixes is given in Table 1.

### 2.2. Binder Characterization

Among the basic physical characterization of WBA and LH, their powder density, specific density, and specific surface were assessed. The powder density was obtained on a gravimetric principle, i.e., from the measurement of dry sample mass and volume. The specific density was determined on the helium pycnometry principle using Pycnomatic ATC (Thermo Scientific, Milan, Italy). The Blaine specific surface was measured according to EN 196-6 [53]. The Brunauer–Emmett–Teller (BET) surface area was tested with an SA 3100 apparatus (Beckman Coulter Life Sciences) using a static dosing method.

Chemical composition of studied binders was measured by X-ray fluorescence analysis (XRF). For the measurement, an Axios sequential WD-XRF spectrometer (PANalytical, Almelo, Netherlands) equipped with an Rh anode end-window X-ray tube fitted with a 50 μm beryllium window was applied. The resulting data were collected by SuperQ software and further evaluated by Omnian software. 

Morphology of WBA was examined using scanning electron microscopy (SEM) with an FEG electron source (Tescan Lyra 3 dual beam microscope, Tescan Brno, s.r.o., Brno, Czech Republic). Elemental composition and mapping were performed using an energy dispersive spectroscopy (EDS) analyzer (X-MaxN) with a 20 mm^2^ silicon drift detector (Oxford instruments, High Wycombe, UK) and AZtecEnergy software (Oxford Instruments). To conduct the measurements, the samples were placed on a carbon conductive tape. SEM and EDS measurements were carried out using a 10 kV electron beam.

X-ray powder diffraction data were collected at room temperature on a Bruker D8 Discoverer powder diffractometer (Bruker AXS GmbH, Karlsruhe, Germany) with parafocusing Bragg–Brentano geometry using CuK_α_ radiation (λ = 0.15418 nm, U = 40 kV, I = 40 mA). Data were scanned over the angular range 5–80° (2θ) with a step size of 0.019° (2θ). Data evaluation was performed in the software package HighScorePlus. 

The particle size distribution was analyzed by an Analysette 22 Micro Tec plus (Fritsch, Idar-Oberstein, Germany) working on a laser diffraction principle. 

As the studied WBA was applied as a mineral admixture, its pozzolanic activity was tested using both Frattini and modified Chapelle tests, for the sake of comparison. The Frattini test was based on a comparison of the Ca(OH)_2_ amount present in the liquid phase in contact with cement with the amount of Ca(OH)_2_ that saturates the environment of equal alkalinity. The measurement was done according to EN 196-5 [54]. The modified Chapelle test was performed using the procedure described in the French standard NF P 18-513 [55]. The results were expressed in mg of Ca(OH)_2_ fixed by 1 g of WBA. 

### 2.3. Methods for Testing Properties of Lime-Based Mortars with WBA Admixing

The rheological performance and workability of fresh mortar mixes was accessed on the basis of a flow table test that was realized according to the standard EN 1015-3 [56].

The hardened mortar specimens were tested at the age of 28 and 365 days (except measurement of hygric and thermal parameters). 

Among structural material characteristics of the hardened mortars, specific density, bulk density, and open porosity were measured. For the tests, 5 specimens were used. The specimens were first dried in a vacuum drier at 60 °C. The specific density *ρ_s_* (kg/m^3^) was obtained by helium pycnometry (see Section 2.2). The dry bulk density *ρ_b_* (kg/m^3^) was determined according to EN 1015-10 [57]. The expanded combined uncertainty of the bulk density test was 1.4%. The relative density and total open porosity *ψ* (%) were calculated based on the bulk density and specific density tests results. The expanded combined uncertainty of the both relative density and total open porosity tests was 2.0%. 

The microstructure of the LH–WBA-based mortars was characterized by the measurement of pore size distribution that was conducted by means of mercury intrusion porosimetry (MIP) using apparatuses Pascal 140 and 440 (Thermo Scientific, Milan, Italy). The dry specimen mass was typically 1–2 g. For the MIP test, three specimens were measured to ensure reproducibility of obtained data. 

Reactivity of WBA in lime-blends was investigated by simultaneous thermal analysis (STA) in the temperature range 50–1000 °C. The DSC (difference scanning calorimetry) and TG (thermogravimetry) curves were recorded simultaneously on a Linseis STA PT1600 TG-DSC/DTA apparatus (Linseis Messgeraete GmbH, Selb, Germany). The STA tests were conducted at a heating rate of 10 °C/min in a dynamic air atmosphere (50 mL/min). The specimen mass was 10–50 mg. The STA experiments were repeated three times due to result reproducibility. 

Mortar mechanical resistance was characterized by compressive strength, flexural strength, and Young’s modulus measurements. The strength tests were performed according to the standard EN 1015-11 [58]. For each mortar mixtures, 5 prismatic specimens with dimensions of 40 × 40 × 160 mm were tested. First, the Young´s modulus *E_d_* (GPa) of samples dried in a vacuum drier at 60 °C was obtained on a dynamic principle using the pulse ultrasonic device DIO 562 (Starmans Electronic, Prague, Czech Republic). The expanded combined uncertainty of this test was 1.7%. The flexural strength *f_f_* (MPa) test was performed in a three point bending test arrangement with span 100 mm. The compressive strength *f_c_* (MPA) was measured on the fragments of specimens from flexural strength testing. The loading area was 40 × 40 mm. The relative expanded uncertainty of these strength tests was 1.4%. 

The evaluation of the compressive strength of the mortars prepared with LH–WBA blends was done by the strength activity index (*SAI*) defined in EN 450-1 [59]. It was calculated as a ratio of the compressive strength of the mortar with partial LH substitution and the compressive strength of the reference mortar MR. 

Hygric and thermal parameters of tested mortars were measured for samples matured to 28 days only. The water absorption coefficient *A_w_* (kg/(m^2^∙s^1/2^)) was measured following EN 1015-18 [60]. The free water intake experiment was performed on 5 specimens. From the measured water absorption coefficient, and saturated moisture content value *w_sat_* (kg/m^3^), the apparent moisture diffusivity *κ* (m^2^/s) was calculated [61]. The expanded combined uncertainty of the water absorption test was 2.3%, and that of the apparent moisture diffusivity test was 3.5%. Water vapor transmission properties as water vapor diffusion coefficient *D* (m^2^/s), water vapor resistance factor *μ* (-), and water vapor permeability *δ* (s), were measured using the cup method under isothermal conditions following standard ISO 12572 [62]. Both the dry-cup and wet-cup arrangements of the test were used. For each tested mortar, 3 specimens for dry-cup test and 3 specimens for wet-cup test were used. The expanded combined uncertainty of the water vapor diffusion test was 2.0% for *D*, 2.8% for *μ*, and 2.1% for *δ*. 

Mortar thermal parameters as thermal conductivity *λ* (W/(m∙K)) and volumetric heat capacity *c* (J/(m^3^∙K)) were obtained by apparatus ISOMET 2114 (Applied Precision, Bratislava, Slovakia) working on a heat impulse measurement technique. The measurement was done at room temperature (23 ± 2) °C on 3 dried specimens and on 3 fully water-saturated specimens. In this way, the boundary values of tested thermal parameters were accessed. The expanded combined uncertainty was 4.3% for the thermal conductivity and 5.1% for the specific heat capacity tests.

### 2.4. Assessment of Environmental Impact of WBA Use as Partial Lime Hydrate Substitution

Due to the limited natural resources for building material production, the environmental assessment of LH–WBA blends was an important issue. In the analysis, carbon footprint and the amount of consumed energy were considered as two basic comparative criteria. The analysis was conducted following an original methodology published by Záleská et al. [63]. The range of the analysis was reduced to the intermediate stage of material processing and the calculations were done for a functional unit of 1 t of a final LH–WBA blend. The lime production stages involved quarrying, extraction, crushing and grinding of raw materials, calcination, hydration, and final grinding. The WBA treatment consisted of grinding only. In analyzed production stages, the storage and transportation were not considered. The energy and emission inventory of the particular production stages used in performed calculations of savings in energy consumption and CO_2_ emission by the use of WBA in lime blends is introduced in Table 2. This data was used on the assumption that electricity was used as the only source of calcination.

The data on emissions and energy usage was compiled from the literature review, as shown in Table 3, and theoretical estimations.

## 3. Results 

### WBA and LH Properties

**Figure 1:**
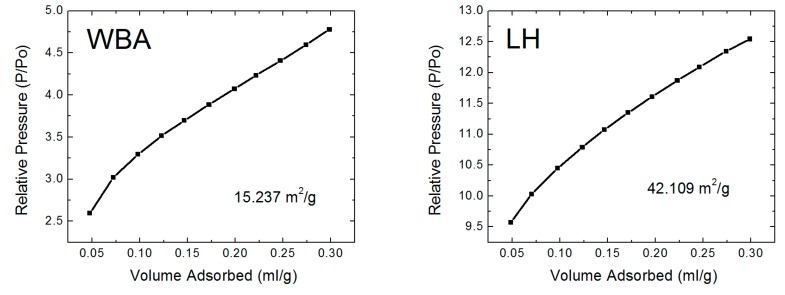
Surface area of WBA and LH measured by a sorption analyzer.

The basic physical characteristics of WBA and LH are summarized in Table 4. The adsorption isotherms are shown in Figure 1. Unfortunately, the tested WBA exhibited significantly lower Blaine fineness compared to LH. Accordingly, the BET surface area of WBA was lower than that of LH. However, as the Blaine fineness of ordinary Portland cement (OPC) usually ranges typically from 300 to 500 m^2^/kg, the Blaine fineness can be considered as good in general, taking into account its intended application as an active mineral admixture. This can be attributed to morphology of WBA and its mechanical activation by milling. The low ratio of powder density to specific density indicated the porous nature of WBA.

The chemical composition of WBA and LH (only main oxides are included) measured by XRF analysis is given in Table 5. 

In the obtained oxide composition of WBA (normalized wt % values are presented), SiO_2_ and Al_2_O_3_ prevailed, which is positive for presumed reactivity of WBA with lime hydrate in the presence of water. Also, high content of CaO was identified in WBA, which is also advantageous for its use as an active admixture with partial hydraulic potential. It should be noted that the oxide composition can also indicate an excessive presence of some salts potentially harmful for lime-based composites, e.g., sulphates, chlorides. Soluble salts are a principal agent of decay of lime rendering and masonry mortars due to their chemical interaction with hydrated products, crystallization, hydration, leaching, efflorescence, and high hygroscopicity. When the chloride solution penetrates lime mortar, it can cause also a loss of calcium hydroxide due to its dissolution. The sulphate induced corrosion (sulphatation) of lime-based materials is in fact transformation of Ca(OH)_2_ to gypsum (CaSO_4_) that is accompanied by the increase in volume of about 17%, which leads to material damage due to crack formation and material disintegration [71]. Moreover, the formation of gypsum decreases significantly the pH value that can destabilize components of lime-pozzolan hydrated compounds [72]. In our case, the amounts of sulphates and chlorides were very low. The maximum limit for SO_3_ content in LH given in EN 459-1 [50] is 2%. As WBA was applied as 5%, 10%, and 15% LH replacement, the limit for maximum SO_3_ content in lime blends was safely achieved. On the other hand, WBA contained a high amount of alkali oxides (Na_2_O + K_2_O), which should be always considered in case of the use of reactive aggregate. The maximum limit for alkali oxides given in EN 450-1 [59] is up to 2 wt % in cement, and up to 5 wt % in fly ash. In our case, the summary content of alkali oxides in WBA was 8.48 wt %, whereas no alkalis were identified in LH. Therefore, the amount of alkalis in all blends of WBA and LH used in this work in mortar compositions may be considered as acceptable and safe. The content of MgO was well below the 4% limit stated in EN 450-1 [59]. 

**Figure 2:**
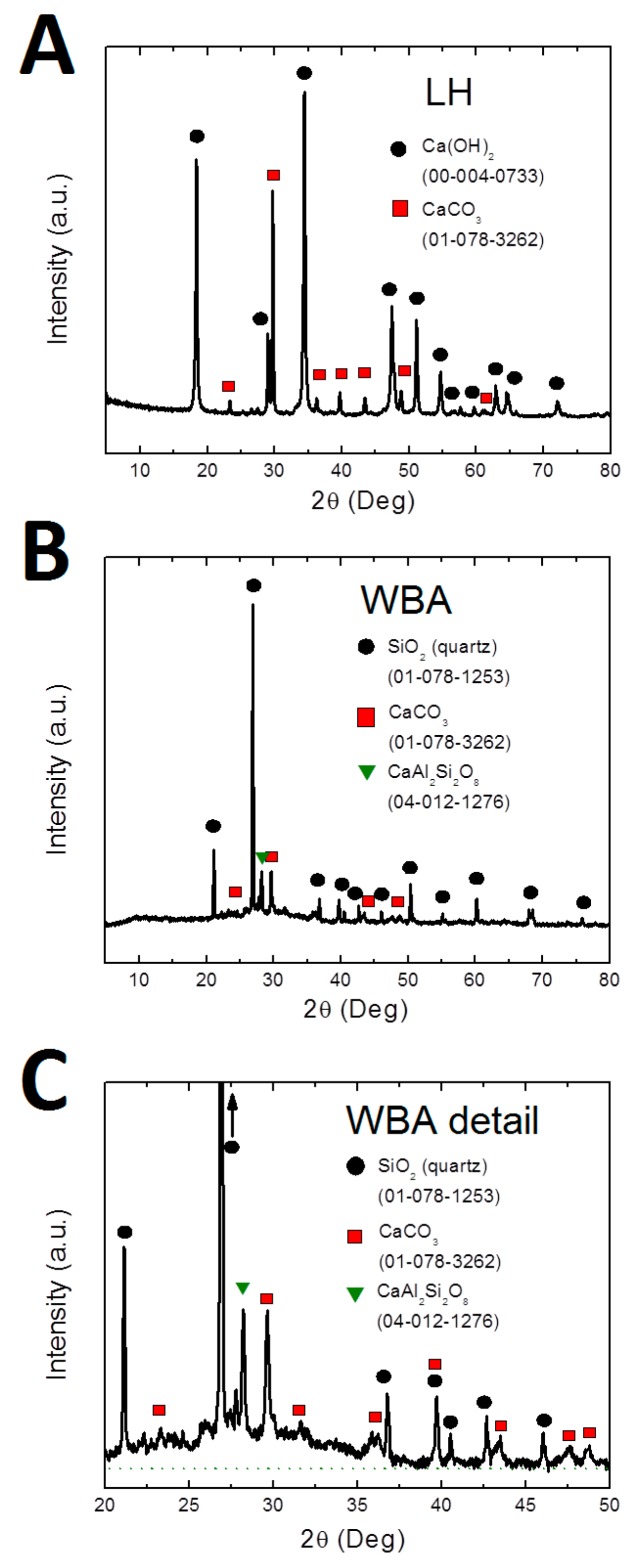
X-ray diffraction patterns. (**A**) LH, (**B**) WBA, (**C**) detail of WBA.

The X-ray diffraction patterns of LH and WBA are shown in Figure 2A–C.

Sample LH contained only calcium hydroxide (approximately 75%, JCPDS 00-004-0733) and calcium carbonate (approximately 25%, JCPDS 01-078-3262). The tested WBA sample contained two major phases: quartz (JCPDS 01-078-1253) and calcium carbonate (JCPDS 01-078-3262). Also, a minor crystalline phase, anorthite (JCPDS 04-012-1276), was identified. Due to the presence of an amorphous phase and very strong diffraction intensity originating from quartz, it was very difficult to accurately calculate ratios of these phases. However, these results confirmed the presence of the major elements, which is in good agreement with XRF analysis results. Contrary to the XRF data, no iron containing substances were observed in XRD patterns.

**Figure 3:**
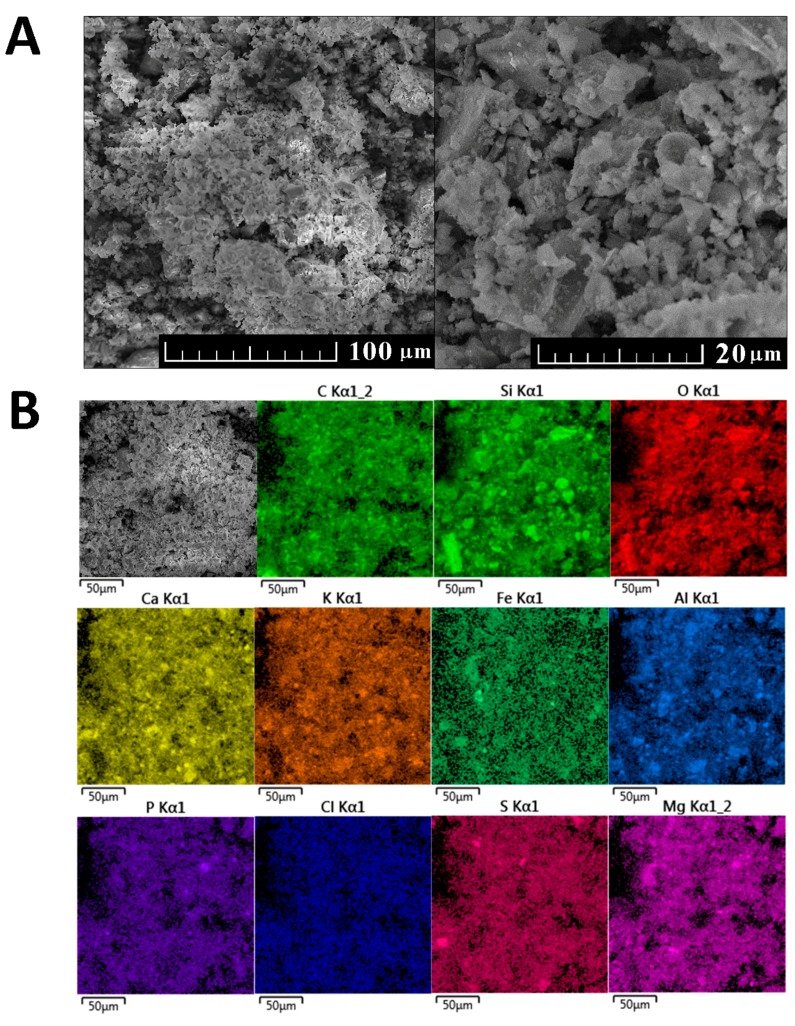
WBA. (**A**) Morphology, (**B**) elemental distribution maps.

The morphology and the chemical composition of WBA were analyzed using scanning electron microscopy and energy dispersive spectroscopy, as shown in see Figure 3A,B. 

**Figure 4:**
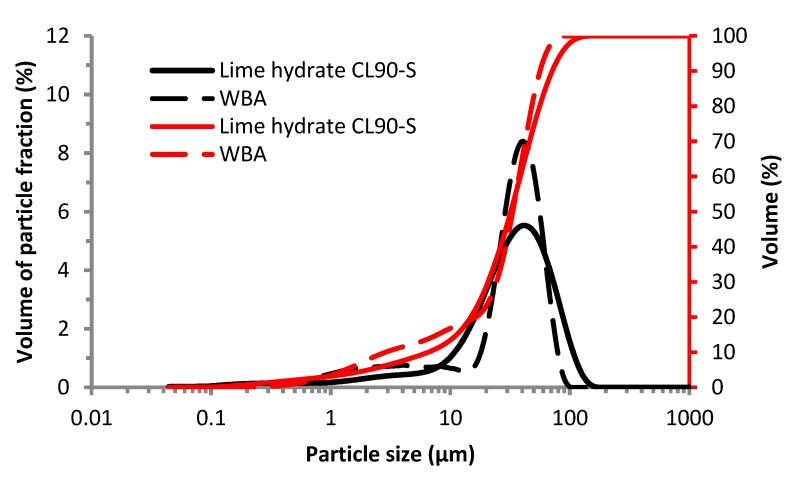
Particle size distribution of WBA and LH: incremental and distribution curves.

The particle size distribution of the mechanically-activated WBA is another very important parameter for pozzolanic activity. It is shown in Figure 4. The grinding resulted in almost similar fineness of WBA as compared to lime hydrate; both powders had the maximum of particle size distribution curves at approximately 40 μm. Slightly worse fineness of WBA was observed in the particle size distribution curve for particles ranging from 7.4 to 29.6 μm only. On the other hand, WBA had a higher volume of fine particles <8.1 μm. As powdered material cannot be pozzolana active if its particles are too large, an upper limit for particle size should be taken into consideration. The recommendations of EN 450-1 [59] set this upper limit to be 45 μm in the form of 40% residue on the 45 μm sieve. WBA met this criteria since it had d_60_ = 38.2 μm as apparent from Figure 4.

The Chapelle test showed fixation of 1294 mg Ca(OH)_2_/g of WBA, which proved its reactivity in lime-based blends. In this sense, the lower limit of 650 mg of Ca(OH)_2_/g of material recommended by Raverdy et al. [73] for a material to be considered pozzolana active was adopted in this paper, which was almost two times higher than the minimum requirement of the Chapelle activity reported by Memon and Khan [74]. The result of the Chapelle test was in agreement with data of the Frattini test as the results obtained for WBA mixes with PC were well below the solubility curve of Ca(OH)_2_ [75]. The pozzolanic activity of WBA we assign to its high fineness and the presence of amorphous silica in its composition.

For the analysis of consistency of fresh mortars, the flow table test was conducted. The results are introduced in Table 6. 

Except for mortar MWBA 5, the increasing content of WBA in the mortar mix led to the increased flow, which was due to the lower Blaine fineness and BET specific surface of WBA compared to LM. These WBA parameters altered the ingress of batch water to binder particles and partially retarded their wetting. The mix with 5 wt % of WBA exhibited lower flow value than obtained for the control mix. It can be attributed to a low content of WBA in the mix composition. Moreover, the difference in the flow value was low. The results of the workability test are very promising for mortar mix design, as a low water/binder ratio can be used without the change in consistency. In this way, with the lower dosage of batch water in the mortar mix, the decrease in porosity can be anticipated, which will be favorable for the improvement of mortars’ mechanical strength and thus their durability.

The structural parameters of examined mortars are given in Table 7. The differences in the total open porosity values were very small, typically in the range of measuring uncertainty. From this point of view, no negative effect of WBA admixing on structural properties of tested mortars was observed. Also, the bulk density and specific density of studied mortars remained almost unaffected by WBA use. The 365 day samples exhibited slightly lower porosity compared to samples cured for 28 days only. This can be attributed to the continuous inner shrinkage of the CH phase, dehydration, and carbonation. Accordingly, with continuous transformation of Ca(OH)_2_ to CaCO_3_, the increase in both specific and bulk densities was apparent. It was due to the bigger molar mass of CaCO_3_ compared to that of Portlandite. From the quantitative point of view, the values of porosity and other structural parameters were typical for such types of lime-based composites. On similar structural characteristics of lime-based mortar reported, e.g., Pachta et al. [76], who received for pure lime mortar made of lime hydrate and sand of siliceous origin (water/binder ratio 0.98) porosity of 30.86% and bulk density of 1650 kg/m^3^.

**Figure 5:**
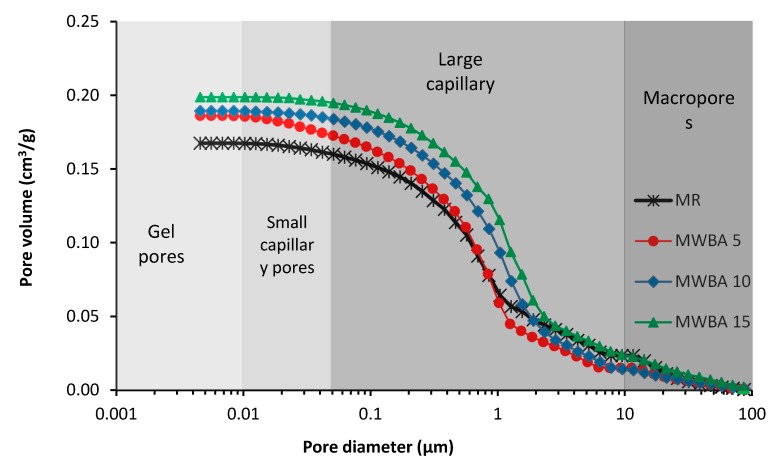
Pore size distribution of tested mortars: 28 day samples.

**Figure 6:**
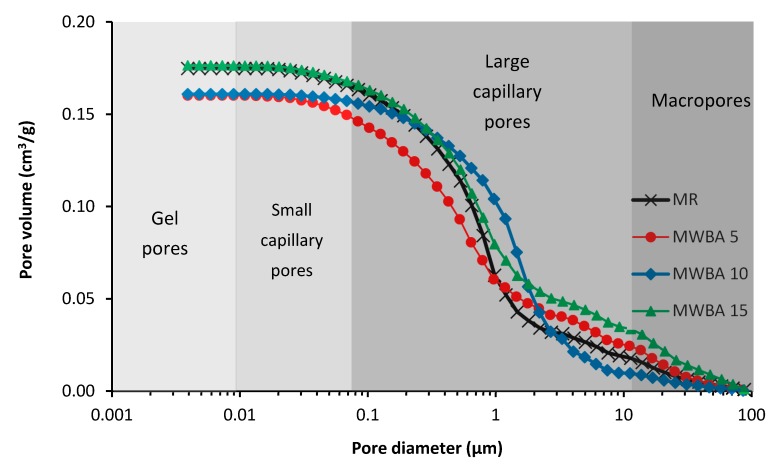
Pore size distribution of tested mortars: 365 day samples.

The results of microstructure analysis obtained by MIP are summarized in Table 8 and shown in Figure 5 and Figure 6. The suffixes in mortar types indicate the samples’ age, i.e., 28 days or 365 days. The classification of pore diameter was done in accordance with classification of pores and features in concrete published Thomas and Jennings in [77]. It must be noted that the threshold pore diameter is an onset point where the pores begin to percolate. Basically, the data on the cumulative pore volume corresponded with the results of structural properties tests given in Table 5. For 28 day aged samples, both the cumulative pore volume and the average pore diameter slightly increased with the increasing amount of WBA dosage in the mortar mix. It was due to the fact that only part of WBA participated in hydration reactions and subsequently in the carbonation process. The 365 day aged samples exhibited typically lower cumulative pore volume and average pore diameter than 28 day samples, whereas material MWBA_365_ 5 achieved even lower total porosity than the control mix, which can be partially attributed to the WBA filler effect. Due to carbonation, the threshold pore dimeter increased for all studied mortar samples. Only small differences between MIP data, as shown in Table 8, and porosity data calculated from the values of basic structural parameters, as shown in Table 7, were observed. They were even within the error bar of the applied methods, especially taking into account that MIP tests were performed for samples having a mass of 1–2 g.

**Figure 7:**
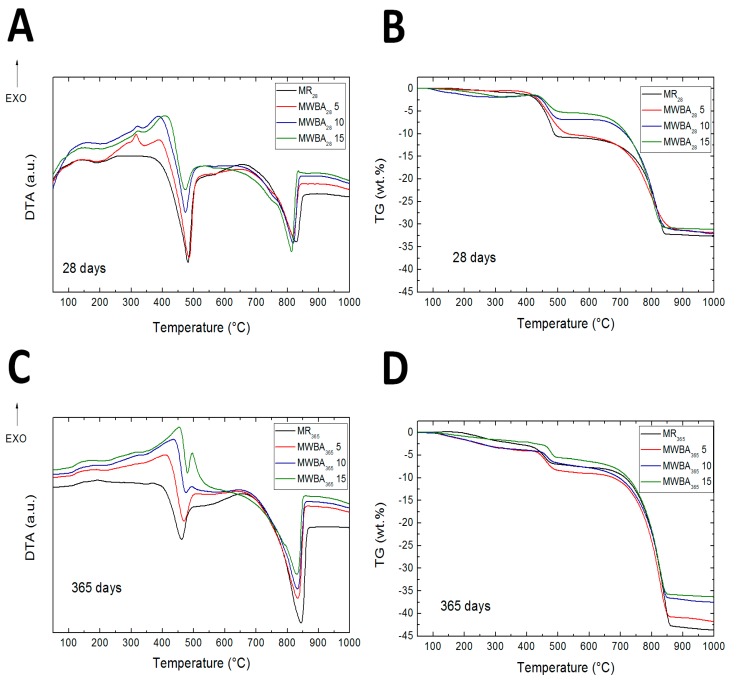
TG and DTA curves of studied mortars. (**A**) DTA for 28 days samples; (**B**) TG for 28 days samples; (**C**) DTA for 365 days samples; (**D**) TG for 365 days samples.

**Figure 8:**
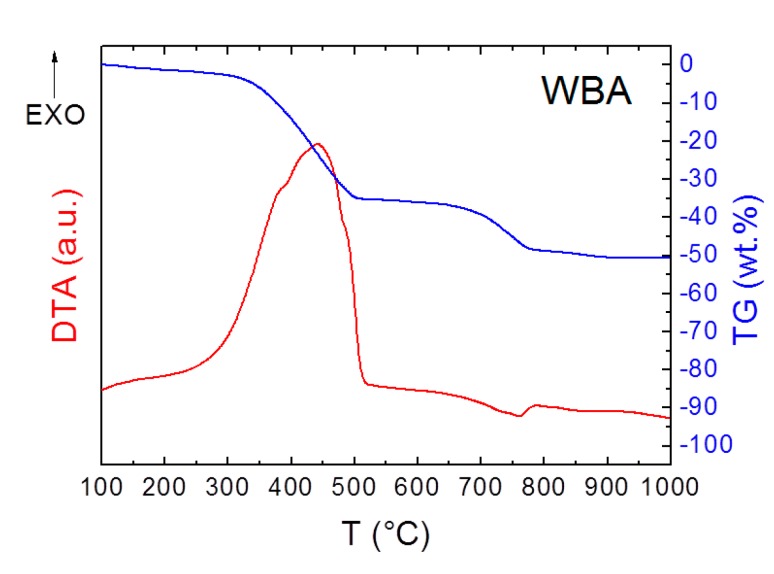
TG and DTA curves of *WBA*.

The STA data of the studied mortar samples is presented in Figure 7. For mortars with WBA-based admixture we identified distinct exothermic process in the temperature interval from 200 to 450 °C. As this peak is not regular for lime-based composites, we performed an additional STA test for WBA only. These results are graphed in Figure 8. Based on differential thermal analysis (DTA) and TG data of WBA, the exothermic reaction in mortars can be assigned to the oxidation of remaining carbon residues in biomass ash. The exothermic effect was accompanied with the weight decrease of approximately 30%. The second, smaller effect can be attributed to the decomposition of calcium carbonate. Similar performance of biomass ash was observed, e.g., Tang et al. [78]. It is obvious the unburnt carbon is always present in biomass ash [79]. Gomez-Barea et al. [80] reported that the carbon present in fly ash is generally present in large amounts, typically 10–60 wt %. Contrary to that, Duan et al. [81] suggested a range of 10–30 wt %. A slight decrease in sample mass in temperature interval from 150 to 250 °C was associated with dehydration of pozzolanic products [30]. The distinct endothermic effect at temperature region 400–530 °C was attributed to the Portlandite dihydroxylation [82]. Weight loss associated with the decarbonation of CaCO_3_ was noted at 650–850 °C. In this temperature range, the second phase of the decomposition of substances formed by pozzolanic reaction of WBA and LH took place also. From the TG curves, it is also obvious that samples after 365 days contained less Portlandite and more calcite in comparison to samples after 28 days.

Based on TG data, Portlandite content in the particular studied mortars was calculated. Calculation was done using the known value of the mass change during the decomposition of pure Ca(OH)_2_, and the mass change of the examined specimen at the corresponding temperature [83]. The relative mass change corresponding to the Portlandite decomposition and Portlandite content is given in Table 9. This data gave evidence of the progress of pozzolanic reaction and carbonation with mortars’ ageing as 365 day samples had Portlandite content considerably lower compared to 28 day matured samples.

Mechanical properties of tested mortars are introduced in Table 10. The mechanical resistance corresponded roughly with structural properties and MIP data. The SAI index calculated as the ratio of the compressive strength of mortars with WBA and the compressive strength of reference mortar was for all studied materials high and gave evidence of the WBA pozzolanity. Moreover, the mechanical parameters of mortars MWBA5 and MWBA10 were for 365 day samples higher than those of control mortar MR. Typically, samples aged 365 days exhibited improved mechanical resistance compared to samples cured for 28 days only. It can be attributed to the progressing carbonation and thus solidification of lime-based matrix and the slow continuous pozzolanic reaction of WBA. According to the standard EN 998-1 [84], mortars can be classified based on their 28 day compressive strength. In Table 10 we proved that the developed mortars can be classified into category CS I (f_c_ in the range 0.4–2.5 MPa). Technical requirements for the replacement of traditional lime-based rendering mortars were summarized by Nogueira et al. [85]. The authors recommended the minimum compressive strength in the range 0.4–2.5 MPa, and flexural strength in the range 0.2–0.7 MPa for mortar samples aged 90 days. Similar minimum values of mechanical parameters recommended for rendering and repointing substitution mortars for ancient buildings were also reported by Veiga et. al [86]. In this respect, the mechanical characteristics of the tested mortars with WBA admixture met the above given criteria for their use as durable and usable alternatives to pure lime-based plasters.

Water transport parameters of studied materials are presented in Table 11. This data was measured for 28 day cured specimens only. The obtained data clearly characterizes the ability of studied mortars to absorb and transport liquid moisture. It should be noted in this respect that the water transport parameters are of particular importance for the practical use of renovation mortars due to their relation to harmful moisture action and water-induced damage. The particular values of studied parameters were in agreement with results of the total porosity and pore size distribution tests, i.e., a slight reduction in water transport was observed for mortar with 5% LH substitution with WBA. Quantitatively, similar moisture diffusivity of mortars was observed, e.g., Pavlíková et al. [87] who analyzed water and salt transport properties of several kinds of commercially produced renovation plasters. The authors stated the pore size distribution is an important parameter in relation to water transport, whereas pores having a diameter above 60 μm are not capillary active. This was not our case, as most of the pores graphed in Figure 5 and Figure 6 were classified as capillary pores, where water molecules were transported using capillary forces.

Data on water vapor transmission properties measured by the cup-method in both dry-cup and wet-cup arrangement is summarized in Table 12. 

The values of water vapor resistance factor were low for all examined mortars, which corresponded with their high porosity. Similar results of the wet-cup test were reported recently, e.g., by Liuzii et al. [88], who obtained for clayed plaster with olive fibers *μ* = 12.5. As the μ-values were for all studied mortars <15, they met the requirement for water vapor transition rates of renovation mortars according to the standard EN 998-1 [84]. From the practical point of view, the obtained water vapor transmission properties ensure possible water evaporation depending on the temperature and moisture conditions of mortar substrate. The ability to evaporate water is one of the features necessary for renovation mortars that are mostly applied on building structures, such as masonry walls, suffering from excessive moisture presence. In this sense, the high water vapor transmission rate may affect the drying behavior of the whole masonry, and therefore may be the decisive factor for its durability as referred by Balen et al. [89].

Thermal properties of studied mortars obtained for dry and fully water saturated specimens are given in Table 13. The thermal conductivity and volumetric heat capacity values were in qualitative agreement with the porosity data and significantly increased due to the moisture presence. This feature must be always considered in practical applications as renovation mortars are often applied on damp masonry. In the case of dry mortar samples, the thermal conductivity decreased with WBA dosage in the mortar mix; the decrease was 2.27% for MWBA 5, 10.2% for MWBA 10, and 13.6% for MWBA 15. For water saturated samples, the decrease in the thermal conductivity was observed for material MWBA 5 only, and it was approximately 9.5%. Due to the slightly higher porosity of mortars MWBA 10 and MWBA 15, their water saturated thermal conductivity increased compared to the control mortar of about 5.4% and 10.4%, respectively. Unlike numerous studies on the testing of thermal parameters of cement composites, investigation of heat transport and storage parameters of lime-based mortars was rarely studied up to now. Barbero-Barrera et al. [90] analyzed thermal conductivity of traditional lime mortar, lime mortars with calcined diatoms, and lime mortars with crushed marble. The thermal conductivity of reference lime mortar and lime mortars with marble aggregate ranged varied from 0.104 to 0.123 W/(m∙K). For mortars with lightweight filler based on calcined diatomite, the authors reported the thermal conductivity in the range from 0.072 to 0.112 W/(m∙K). Stefanidou et al. [91] measured the thermal conductivity of lime composites with pozzolans of different origins in the range 0.16–0.39 W/(m∙K). When the results obtained for mortars with WBA as mineral admixture are compared with those published in the above given literature, the measured thermal conductivities were higher than those of other authors. On the other hand, the thermal conductivity of MWBA materials was much lower than values obtained for PC/pozzolan mortar by Pavlíková et al. [75]. Moreover, as required by EN 998-1 [86] and EN 1745 [92], the thermal conductivity of examined mortars met criteria for renovation mortars.

The results of the environmental impact assessment of WBA use in mortar composition are given in Table 14. 

The data on the savings of energy consumption and CO_2_ emission by the use of WBA in lime blends proved the eco-efficiency of the newly developed mortars taking into consideration their functional and structural parameters, as shown in Table 6, Table 7, Table 8, Table 9, Table 10, Table 11, Table 12 and Table 13, which were close to those obtained for control lime mortar.

## 4. Conclusions

Wood-based biomass ash, due its suitable chemical and mineralogical composition and particle size distribution that was similar to commercially delivered lime hydrate, was studied as a possible pozzolana active admixture in the composition of lime-based mortars for renovation purposes. Based on a complex chemical, physical, and mineralogical analysis of mechanically-activated WBA and detailed testing of structural, mechanical, hygric, and thermal properties of newly developed mortars with incorporated WBA as a constituent of blended binder, the following conclusions were drawn:

- The Chapelle test, STA, and assessment of the strength activity index proved high pozzolanic activity of WBA;
- Admixing of WBA had no negative influence of fresh mix consistency and workability that was even improved for mortars with higher dosage of WBA;
- Mortars’ structural parameters, such as the bulk density, specific density, and total open porosity, remained almost unaffected by WBA use;
- Prolonged curing yielded the decreased porosity and, on the contrary, increased both the specific and bulk densities due to the continuous inner shrinkage of the CH phase, dehydration, carbonation, and progress of the pozzolanic reaction;
- Mechanical parameters of mortars with WBA met the criteria for their use as durable and usable alternatives to traditional lime-based rendering mortars;
- Water transport parameters of tested mortars were unaffected by WBA use and proved a high rate of water transport in their porous structure. This mortar behavior can be beneficially used in desalination of salt contaminated buildings; in the case of the use of the developed materials for renovation purposes, they must be enriched with water repellents in order to reduce moisture ingress;
- The water vapor transition rate of all tested mortars met the requirements imposed on renovation mortars;
- Both the thermal conductivity and specific heat capacity values decreased with a higher dosage of WBA in the mortar mix. Moreover, the thermal conductivity of examined mortars met criteria for renovation mortars;
- The environmental assessment showed a decrease in both carbon dioxide production and energy consumption with the increasing WBA content in mortar dry mix.

In summary, the functional properties of developed mortars with WBA dosages were good in general, pointing to their presumed application as rendering and walling renovation mortars replacing traditional pure lime-based materials. In addition, the products of pozzolanic reaction of WBA and LH are more stable and durable in the presence of excessive moisture and salt action than lime hydrates and carbonates. Calcium carbonate is also well dissoluble by strong acids formed by the reaction of acid-forming atmospheric pollutants with water, which is not the case of CSH (calcium-silicate-hydrate) and CASH (calcium-aluminum-silicate-hydrate) phases. From the practical point of view, blending of WBA and lime-hydrate gave a binder acceptable by cultural heritage authorities and building specialists for repair of historical buildings and monuments, where use of PC is a wrong choice. Nevertheless, in formulation of the final mortar mix with incorporated WBA as a pozzolan admixture, the addition of water repellents, workability, adhesive, and other additives to adjust its properties for specific rendering and structural purposes must be considered and experimentally evaluated. Above all, additives selected on the basis of their properties and historical use should be preferred. These should include organic additives, such as polysaccharides, proteins, and fatty acids and oils. 

The tested WBA after its mechanical activation represents alternative pozzolana active powdered admixture for the production of lime-based eco-efficient and sustainable building composites. The environmental contribution of WBA use in mortar composition is quite apparent both from the point of view of limited natural resources for production of building materials and reuse of biomass ash as a by-product of sustainable energy production. Also, reduction of pollution coming from the lime industry, and energy savings by the use of WBA should be highlighted in this respect.

## Figures and Tables

**Table 1:** Composition of studied mortars, mass (g). WBA: wood-based biomass ash; MR: reference lime mortar.

Mortar Mix	Lime Hydrate	WBA	Silica Sand	Water	w/b (-)
MR	1350.0	-	3 × 1350	1350	1.0
MWBA 5	1282.5	67.5	3 × 1350	1350	1.0
MWBA 10	1215.0	135.0	3 × 1350	1350	1.0
MWBA 15	1147.5	202.5	3 × 1350	1350	1.0

**Table 2:** Energy and emission inventory of the production stages (per ton of Ca(OH)_2_ or WBA).

Material/Production Process	Energy Consumption (kWh)	CO_2_ Emission (kg)
Lime hydrate		
quarrying	10.3	2.5
crushing and grinding	1.46	0.8
calcination	37.5	721
hydration	13.1	7.4
grinding	22.1	12.1
**WBA**		
grinding	22.1	12.1

**Table 3:** Energy and emission data sources.

Material	Specification	Analysis Range	Key References
Lime hydrate	Product	Mining, grinding, calcination, hydration	[64,65,66,67]
WBA	Waste product	Grinding	[68,69,70]

**Table 4:** Physical parameters of WBA and lime hydrate (LH). BET: Brunauer–Emmett–Teller.

Material	Blaine Fineness (m^2^/kg)	BET Surface Area (m^2^/kg)	Specific Density (kg/m^3^)	Powder Density (kg/m^3^)
LH	2211	42,109	2210	239
WBA	781	15,237	2557	875

**Table 5:** X-ray fluorescence analysis (XRF) chemical composition of WBA and LH.

Substance	WBA	LH
	Amount (wt %)	
CuO	0.01	-
Na2O	0.92	-
ZnO	0.13	-
MgO	3.18	0.54
Al_2_O_3_	10.32	-
BaO	0.16	-
SiO_2_	45.57	0.01
P_2_O_5_	2.63	-
SO_3_	3.24	0.07
SrO	0.07	0.03
Cl	0.64	-
ZrO_2_	0.04	-
K_2_O	7.56	-
CaO	18.08	99.33
TiO_2_	0.82	-
V_2_O_5_	0.02	-
PbO	0.02	-
Cr_2_O_3_	0.02	-
MnO	1.85	-
Fe_2_O_3_	4.71	0.02
∑	100.00	100.00

**Table 6:** Workability of fresh mortars.

Material	Flow (mm)
MR	185/180
MWBA 5	180/180
MWBA 10	220/220
MWBA 15	245/250

**Table 7:** Basic structural characteristics of studied mortars.

Material	28 Day Samples			365 Day Samples		
	*ρ_b_* (kg/m^3^)	*ρ_s_* (kg/m^3^)	*ψ* (%)	*ρ_b_* (kg/m^3^)	*ρ_s_* (kg/m^3^)	*ψ* (%)
MR	1742	2578	32.4	1766	2614	32.4
MWBA 5	1745	2578	32.3	1773	2603	31.9
MWBA 10	1739	2577	32.6	1772	2605	31.9
MWBA 15	1736	2579	32.7	1761	2606	32.4

**Table 8:** Pore size distribution parameters accessed by mercury intrusion porosimetry (MIP).

Mortar Type	Cumulative Pore Volume (cm^3^/g)	Average Pore Diameter (μm)	Threshold Pore Diameter (μm)	Total Porosity (%)
MR_28_	0.1674	0.960	0.835	31.3
MR_365_	0.1705	0.791	1.468	30.9
MWBA_28_ 5	0.186	0.958	1.247	31.6
MWBA_365_ 5	0.1599	0.791	1.444	30.8
MWBA_28_ 10	0.1892	1.036	1.904	32.4
MWBA_365_ 10	0.1602	1.041	2.677	31.5
MWBA_28_ 15	0.1906	1.041	2.306	33.1
MWBA_365_ 15	0.1750	1.038	1.796	32.3

**Table 9:** TG mass change and Portlandite content.

Mortar Type	Mass Change (%)		Portlandite Content (wt %)	
	28 Days	365 Days	28 Days	365 Days
MR	9.3	4.2	38.21	17.25
MWBA 5	9.2	4.0	37.8	16.44
MWBA 10	5.4	2.5	22.19	10.27
MWBA 15	3.7	2.4	15.20	9.87

**Table 10:** Mechanical parameters of studied mortars. SAI: strength activity index.

Material	*f_f28_* (MPa)	*f_f365_* (MPa)	*f_c28_* (MPa)	*f_c365_* (MPa)	*E_d28_* (GPa)	*E_d365_* (GPa)	*SAI_28_* (%)	*SAI_365_* (%)
MR	0.79	0.90	1.08	2.33	2.5	2.8	-	-
MWBA 5	0.72	1.23	0.87	2.88	2.7	3.6	80.6	123.6
MWBA 10	0.65	1.15	0.85	2.40	2.6	3.4	78.7	103.0
MWBA 15	0.61	0.89	0.81	2.12	2.4	2.9	75.0	91.0

**Table 11:** Water transport parameters of studied mortars.

Material	*A_w_* (kg/(m^2^∙s^1/2^))	*w_sat_* (kg/m^3^)	*κ* (× 10^−7^ m^2^/s)	*κ* Difference from MR (%)
MR	0.307	323.2	9.0	-
MWBA 5	0.304	320.7	8.9	−1.1
MWBA 10	0.310	325.2	9.1	1.1
MWBA 15	0.321	327.2	9.6	6.7

**Table 12:** Water vapor transmission parameters.

**Dry-Cup**				
**Material**	***δ*** **(× 10^−11^ s)**	***D*** **(× 10^−6^ m^2^/s)**	***μ*** **(-)**	***μ*** **Difference from MR (%)**
MR	1.50	2.05	12.2	-
MWBA 5	1.48	2.03	12.3	0.8
MWBA 10	1.52	2.08	12.0	−1.6
MWBA 15	1.62	2.21	11.3	−9.8
**Wet-cup**				
MR	1.71	2.34	10.7	-
MWBA 5	1.66	2.27	11.0	2.8
MWBA 10	1.79	2.45	10.2	−4.7
MWBA 15	1.99	2.72	9.2	−14.0

**Table 13:** Heat transport and storage properties.

Material	*λ_dry_* (W/(m∙K))	*c_vdry_* (× 10^6^ J/(m^3^∙K))	*λ_sat_* (W/(m∙K))	*c_vsat_* (× 10^6^ J/(m^3^∙K))
MR	0.88	1.44	2.22	1.62
MWBA5	0.86	1.59	2.01	1.64
MWBA10	0.79	1.43	2.34	1.70
MWBA15	0.76	1.39	2.45	1.71

**Table 14:** Savings in energy consumption and CO_2_ emission by the use of WBA in lime blends (per ton of LH–WBA blend).

Material	Energy Consumption (kWh)	CO_2_ Emission (kg)
LH–WBA 5	0.8	5.9
LH–WBA 10	1.6	11.8
LH–WBA 15	2.4	17.6

## References

[1] Gris E.R., Paine K.A., Heath A., Norman J., Pinder H. (2013). Compressive strength development of binary and ternary lime-pozzolan mortars. Mater. Des..

[2] Callebaut K., Elsen J., Van Balen K., Viane W. (2001). Nineteenh century hydraulic restoration mortars in the Saint Michael’s Church (Leuven, Belgium) Natural hydraulic lime or cement?. Cem. Concr. Res..

[3] Lea F.M. (1976). The Chemistry of Cement and Concrete.

[4] Ponce-Anton G., Arizzi A., Zuluaga M.C., Cultrone G., Ortega L.A., Mauleon J.A. (2019). Mineralogical, textural and physical characterization to determine deterioration susceptibility of Irulegi castle lime mortars (Navarre, Spain). Materials.

[5] Jonaitis B., Antonovic V., Sneideris A., Boris R., Zavalis R. (2019). Analysis of physical and mechanical properties of the mortar in the historic retaining wall of the Gediminas Castle Hill (Vilnius, Lithuania). Materials.

[6] Izzo F., Grifa C., Germinario C., Mercurio M., De Bonis A., Tomay L., Langella A. (2018). Production technology of mortar-based building materials from the Arch of Trajan and the Roman Theatre in Benevento, Italy. Eur. Phys. J. Plus.

[7] Dalto D.P.D., Ribeiro R.C.D., de Moura L.C.R. (2018). Characterization of the lime mortars of the Rui Barbosa House Museum in Rio De Janeiro, Brazil. Minerals.

[8] Sepulcre-Aguilar A., Hernández-Olivares F. (2010). Assessment of phase formation in lime-based mortars with added metakaolin, Portland cement and sepiolite, for grouting of historic masonry. Cem. Concr. Res..

[9] Mosquera M.J., Silva B., Prieto B., Ruiz-Herrera E. (2006). Addition of cement to lime based mortars: Effect on pore structure and vapor transport. Cem. Concr. Res..

[10] Faria-Rodrigues P., Henriques F.M.A. (2004). Current mortars in conservation: An overview. Restor. Build. Monum..

[11] Elert K., Rodriguez-Navarro C., Pardo E.S., Hansen E., Cazalla O. (2002). Lime mortars for the conservation of historic buildings. Stud. Conserv..

[12] Torres I., Matias G. (2016). Sustainable mortars for rehabilitation of old plasters. Eng. Struct..

[13] Barbero-Barrera M., Maldonado-Ramos L., Van Balenb K., García-Santosa A., Neila-González F. (2014). Lime render layers: An overview of their properties. J. Cult. Herit..

[14] Schueremans L., Cizer Ö., Janssens E., Serré G., Van Balen K. (2011). Characterization of repair mortars for the assessment of their compatibility in restoration projects: Research and practice. Constr. Build. Mater..

[15] Arizzi A., Viles H., Cultrone G. (2012). Experimental testing of the durability of lime-based mortars used for rendering historic buildings. Constr. Build. Mater..

[16] Farina P., Henriques F., Rato V. (2008). Comparative evaluation of lime mortars for architectural conservation. J. Cult. Herit..

[17] Govaerts Y., Hayen R., de Bouw M., Verdonck A., Maulebroeck W., Mertens S. (2018). Performance of a lime-based insulating render for heritage buildings. Constr. Build. Mater..

[18] Vitruvius P. (2012). Vitruvius: The Ten Books of Architecture.

[19] Veiga M.R., Santos Silva A., Tavares M., Santos A.R., Lampreia N. (2013). Characterization of renders and plasters from a 16th century Portuguese military structure: Chronology and durability. Restor. Build. Monum..

[20] Santos Silva A., Cruz T., Paiva M.J., Candeias A., Adriano P., Schiavon N., Mirão J. (2011). Mineralogical and chemical characterization of historical mortars from military fortifications in Lisbon harbour (Portugal). Environ. Earth Sci..

[21] Veiga R. (2017). Air lime mortars: What else do we need to know to apply them in conservation and rehabilitation interventions? A review. Constr. Build. Mater..

[22] Barnat-Hunek D., Siddigue R., Klimek B., Franus M. (2017). The use of zeolite, lightweight aggregate and boiler slag in restoration renders. Constr. Build. Mater..

[23] Ozen S., Goncuoglu M.C., Liguouri B., de Gennaro B., Cappeletti P., Gatta G.D., Iucolano F., Colella C. (2016). A comprehensive evaluation of sedimentary zeolites from Turkey as pozzolanic addition of cement- and lime-based binders. Constr. Build. Mater..

[24] Pavlík V., Užáková M. (2016). Effect of curing conditions on the properties of lime, lime-metakaolin and lime-zeolite mortars. Constr. Build. Mater..

[25] Martínez W., Alonso-Guzman E.M., Rubio J.C., Bedolla J.A., Velasco F.A., Torres A.A. (2008). Handmade hydrated lime mechanical motors mehavior, added with cactus sap and volcanic ash, for their use in colonial monument restoration and conservation. Rev. Constr..

[26] Sala E., Giustina I., Plizzari G.A., D’Ayala D., Fodde E. (2008). Lime Mortar with Natural Pozzolana: Historical Issues and Mechanical Behavior, Structural Analysis of Historic Construction.

[27] Nozahic V., Amziane S., Torrent G., Saidi K., De Baynast H. (2012). Design of green concrete made of plant-derived aggregates and a pumice-lime binder. Cem. Concr. Res..

[28] Sierra E.J., Miller S.A., Sakulich A.R., MacKenzie K., Barsoum M.W. (2010). Pozzolanic activity of diatomaceous earth. J. Am. Ceram. Soc..

[29] Loganina V.I., Pyshkina I.S., Martyashin G.V. (2017). Effect of the supplement based on calcium hydrosilicates on the resistance of lime coatings. Mag. Civ. Eng..

[30] Gameiro A., Silva A.S., Faria P., Grilo J., Branco T., Veiga R., Velosa A. (2014). Physical and chemical assessment of lime–metakaolin mortars: Influence of binder: Aggregate ratio. Cem. Concr. Compos..

[31] Cachim P., Velosa A.L., Rocha F. (2010). Effect of Portuguese metakaolin on hydraulic lime concrete using different curing conditions. Constr. Build. Mater..

[32] Bulut Ü. (2010). Use of perlite as a pozzolanic addition in lime mortars. GU J. Sci..

[33] Bras A., Henriques F.M.A., Cidade M.T. (2010). Effect of environmental temperature and fly ash addition in hydraulic lime grout behavior. Constr. Build. Mater..

[34] Nayaka R.R., Alegaram U.J., Jumaat M.Z., Yusoff S.B. (2018). Microstructural investigation and durability performance of high volume industrial by-products-based masonry mortars. Constr. Build. Mater..

[35] Bediako M., Kevern J.T., Dodoo-Arhin D. (2017). Co-fired Ghanaian clay-palm kernel shells pozzolan: Thermogravimetric, Si-29 and Al-27 MA NMR characteristics. Constr. Build. Mater..

[36] Cordeiro G.C., Toledo Filho R.G., Tavares L.M., Fairbairn E.M.R. (2008). Pozzolanic activity and filler effect of sugar cane bagasse ash in Portland cement and lime mortars. Cem. Concr. Compos..

[37] Pavia S., Walker R., Veale P., Wood A. (2014). Impact of the properties and reactivity of rice husk ash on lime mortar properties. J. Mater. Civ. Eng..

[38] Cortina M.G., Dominguez L.D., de Madrid E.U.A.T., de Madrid E.T.S.A. (2002). Aired-lime and chamotte hydraulic mortars. Mater. Constr..

[39] Bakolas A., Aggelakopoulou E., Moroupoulou A. (2008). Evaluation of pozzolanic activity and physico-mechanical characteristics in ceramic powder-lime pastes. J. Therm. Anal. Calorim..

[40] Xu S.Q., Ma Q.L., Wang J.L., Wang L.L. (2018). Grouting performance improvement for natural hydraulic lime-based grout via incorporating silica fume and silicon-acrylic latex. Constr. Build. Mater..

[41] Zhnag D.J., Zhao J.H., Wang D.M., Xu C.Y., Zhai M.Y., Ma X.D. (2018). Comparative study on the properties of three hydraulic lime mortar systems: Natural hydraulic lime mortar, cement-aerial lime-based mortar and slag-aerial lime-based mortar. Constr. Build. Mater..

[42] Pavia S., Regan D. (2010). Influence of cement kiln dust on the physical properties of calcium lime mortars. Mater. Struct..

[43] Mar Barbero-Barrera M., Flores Medina N., Guardia-Martin C. (2017). Influence of the addition of waste graphite powder on the physical and microstructural performance of hydraulic lime pastes. Constr. Build. Mater..

[44] Silva F.C., Cruz N.C., Tarelho L.A.C., Rodrigues S.M. (2019). Use of biomass ash-based materials as soil fertilisers: Critical review of the existing regulatory framework. J. Clean. Prod..

[45] Directive 2009/28EC, Official Journal of the European Union. https://eur-lex.europa.eu/eli/dir/2009/28/oj.

[46] Katare V.D., Madurwar M.V. (2019). Use of processed biomass ash as a sustainable pozzolana. Curr. Sci..

[47] Martirena F., Monzo J. (2018). Vegetable ashes as Supplementary Cementitious Materials. Cem. Concr. Res..

[48] Jankovský O., Pavlíková M., Sedmidubský D., Bouša D., Lojka M., Pokorný J., Záleská M., Pavlík Z. (2017). Study on pozzolana activity of wheat straw ash as potential admixture for blended cements. Ceram. Silik..

[49] Teixeira E.R., Mateus R., Camões A., Branco F.G. (2019). Quality and durability properties and life-cycle assessment of high volume biomass fly ash mortar. Constr. Build. Mater..

[50] (2015). EN 459-1, Building Lime—Part 1: Definitions, Specifications and Conformity Criteria.

[51] (1998). EN 1015-2, Methods of Test for Mortar for Masonry—Part 2: Bulk Sampling of Mortars and Preparation of Test Mortars.

[52] (2016). EN 196-1, Methods of Testing Cement—Part 1: Determination of Strength.

[53] (2010). EN 196-6, Methods of Testing Cement—Part 6: Determination of Fineness.

[54] (2011). EN 196-5, Methods of Testing Cement—Part 5: Pozzolanicity Test for Pozzolanic Cement.

[55] (2009). NF P 18-513, 2009. Pozzolanic Addition for Concrete—Metakaolin: Definitions, Specifications and Conformity Criteria, Annex A.

[56] (1999). EN 1015-3, Methods of Test for Mortar for Masonry—Part 3: Determination of Consistence of Fresh Mortar (by Flow Table).

[57] (1999). EN 1015-10, Methods of Test for Mortar for Masonry—Part 10: Determination of Dry Bulk Density of Hardened Mortar.

[58] (1999). EN 1015-11, Methods of Test for Mortar for Masonry—Part 10: Determination of Flexural and Compressive Strength of Hardened Mortar.

[59] (2012). EN 450-1, Fly Ash for Concrete—Part 1: Definition, Specifications and Conformity Criteria.

[60] (2002). EN 1015-18, Methods of Test for Mortar for Masonry—Part 18: Determination of Water-Absorption Coefficient Due to Capillary Action of Hardened Mortar.

[61] Kumaran M.K. (1999). Moisture diffusivity of building materials from water absorption measurements. J. Therm. Envel. Build. Sci..

[62] (2001). ISO 12572, Hygrothermal Performance of Building Materials and Products—Determination of Water Vapour Transmission Properties.

[63] Záleská M., Pavlík Z., Pavlíková M., Scheinherrová L., Pokorný J., Trník A., Svora P., Fořt J., Jankovský O., Suchorab Z. (2018). Biomass ash-based mineral admixture prepared from municipal sewage sludge and its application in cement composites. Clean Technol. Environ..

[64] Kittipongvises S. (2017). Assessment of environmental impacts of limestone quarrying operations in Thailand. Environ. Clim. Technol..

[65] Sagastume Gutiérrez A., Van Caneghem J., Cogollos Martínez J.B., Vandecasteele C. (2012). Evaluation of the environmental performance of lime production in Cuba. J. Clean. Prod..

[66] Alcántara V., Cadavid Y., Sánchez M., Uribe C., Echeverri-Uribe C., Morales J., Obando J., Amell A. (2018). A study case of energy efficiency, energy profile, and technological gap of combustion systems in the Colombian lime industry. Appl. Therm. Eng..

[67] European Commission 2001 Integrated Pollution Prevention and Control (IPPC), Reference Document on Best Available Techniques in the Cement and Lime Manufacturing Industries. http://www.epa.ie/downloads/advice/brefs/cement.pdf.

[68] Pavlík Z., Fořt J., Záleská M., Pavlíková M., Trník A., Medved I., Keppert M., Koutsoukos P.G., Černý R. (2016). Energy-efficient thermal treatment of sewage sludge for its application in blended cements. J. Clean. Prod..

[69] Valderrama C., Granados R., Cortina J.L., Gasol C.M., Guillem M., Josa A. (2013). Comparative LCA of sewage sludge valorisation as both fuel and raw material substitute in clinker production. J. Clean. Prod..

[70] Xu C.H., Chen W., Hong J. (2014). Life-cycle environmental and economic assessment of sewage sludge treatment in China. J. Clean. Prod..

[71] Tittarelli F., Moriconi G., Bonazza A. (2008). Atmospheric deterioration of cement plaster in a building exposed to a urban environment. J. Cult. Herit..

[72] Pavlíková M., Pokorný J., Jankovský O., Záleská M., Vavro M., Souček K., Pavlík Z. (2018). The effect of the sodium sulphate solution exposure on properties and mechanical resistance of different kinds of renders. Ceram. Silik..

[73] Raverdy M., Brivot F., Paillére A.M., Dron R. (1980). Appréciation de I’Activité Pouzzolanique des Constituants Secondaires. Proceedings of the 7th International Congress on the Chemistry of Cement.

[74] Memon S.A., Khan M.K. (2018). Ash blended cement composites: Eco-friendly and sustainable option for utilization of corncob ash. J. Clean. Prod..

[75] Pavlíková M., Zemanová L., Pokorný J., Záleská M., Jankovský O., Lojka M., Sedmidubský D., Pavlík Z. (2018). Valorization of wood chip ash as an eco-friendly mineral admixture in mortar mix design. Waste Manag..

[76] Pachta V., Triantafyllaki S., Stefanidou M. (2018). Performance of lime-based mortars at elevated temperatures. Constr. Build. Mater..

[77] Thomas J., Jennings H. The Science of Concrete. http://iti.northwestern.edu/cement/index.html.

[78] Tang Z.J., Fang P., Huang J.H., Tang Z.X., Cen C.P. (2015). Investigation on thermodynamics characteristics of biomass thermal decomposition using TG/DSC method. Proceedings of the 5th International Conference on Advanced Design and Manufacturing Engineering.

[79] James A.K., Thring R.W., Helle S., Ghuman H.S. (2012). Ash management review—Applications of biomass bottom ash. Energies.

[80] Gomez-Barea A., Vilches L., Campoy M., Fernandez-Pereira C. (2009). Plant optimization and ash recycling in fluidised waste gasification. Chem. Eng. J..

[81] Duan L., Liu D., Chen X., Zhao C. (2012). Fly ash recirculation by bottom feeding on a circulating fluidized bed boiler co-burning coal sludge and coal. Appl. Energy.

[82] Ciyer Ö., Rodriguez-Navarro C., Ruiz-Agudo E., Elsen J., Van Gemert D., Van Balen K. (2012). Phase and morphology evolution of calcium carbonate precipitated by carbonation of hydrated lime. J. Mater. Sci..

[83] Dweck J., Buchler P.M., Coelho A.C.V., Cartledge F.K. (2000). Hydration of a Portland cement blended with calcium carbonate. Thermochim. Acta.

[84] (2016). EN 998-1, Specification for Mortar for Masonry—Part 1: Rendering and Plastering Mortar.

[85] Nogueira R., Ferreira Pinto A.P., Gomes A. (2018). Design and behaviour of traditional lime-based plasters and renders. Review and critical appraisal of strengths and weaknesses. Cem. Concr. Compos..

[86] Veiga M., Aguiar J., Carvalho S.A.S.F., Lourenço P., Roca P. (2001). Methodologies for characterization and repair of mortars of ancient buildings. Historical Constructions.

[87] Pavlíková M., Pavlík Z., Keppert M., Černý R. (2011). Salt transport and storage parameters of renovation plasters and their possible effects on restored buildings’ walls. Constr. Build. Mater..

[88] Liuzzi S., Rubino C.H., Stefanizzi P., Petrella A., Boghetich A., Casavola C., Pappalettera G. (2018). Hygrothermal properties of clayed plasters with olive fibers. Constr. Build. Mater..

[89] Van Balen K., Papayianni I., Van Hess R., Binda L., Waldum A. (2005). Introduction to requirements for and functions and properties of repair mortars. Mater. Struct..

[90] Barbero-Barrera M.M., García-Santos A., Neila-González F.J. (2014). Thermal conductivity of lime mortars and calcined diatoms. Parameters influencing their performance and comparison with the traditional lime and mortars containing crushed marble used as renders. Energy Build..

[91] Stefanidou M., Assael M., Antoniadis K., Matziaroglou G. (2010). Thermal conductivity of building materials employed in the preservation of traditional structures. Int. J. Thermophys..

[92] (2012). EN 1745, Masonry and Masonry Products—Methods for Determining Thermal Properties.

